# Network-based toxicological analysis of core targets and pathways of bisphenol A-driven hepatocellular carcinoma

**DOI:** 10.1016/j.bbrep.2026.102563

**Published:** 2026-04-02

**Authors:** Yanan Zhang, Yang Wu, Chujiang Wu, Yuxin He, Yujie Zhai, Zihan Zhou, Jiucong Zhang, Xiaofeng Zheng

**Affiliations:** a Department of Gastroenterology, the 940th Hospital of Joint Logistic Support Force of PLA, Lanzhou, Gansu, 730050, China; b The First Clinical Medical College of Gansu University of Chinese Medicine, Lanzhou, Gansu, 730000, China; c General Practice Department, Pengzhou Jiuchi Town Hospital, Chengdu, Sichuan, 611930, China; d Department of Medicine, Northwest Minzu University, Lanzhou, Gansu, 730030, China; e Department of Gastroenterology, the Second Hospital of Lanzhou University, Lanzhou, Gansu, 730030, China

**Keywords:** Network toxicology, Bisphenol A, Hepatocellular carcinoma, Molecular docking

## Abstract

**Background:**

Bisphenol A (BPA), an industrial compound widely used in plastics and food packaging, has been implicated in the development of various diseases. This study aims to elucidate the molecular toxicity mechanisms of BPA in hepatocellular carcinoma (HCC), providing a theoretical foundation for the prevention and treatment of HCC.

**Methods:**

By retrieving BPA and HCC-associated genes from multiple databases and identifying their intersections, we performed protein-protein interaction (PPI) analysis and visualization of the intersecting genes. Potential mechanisms were explored through Gene ontology (GO) and kyoto encyclopedia of genes and genomes (KEGG) pathway enrichment analysis. Core genes were identified using the Degree algorithm. Their expression levels in HCC were validated using the TCGA database, and Kaplan-Meier survival curves were constructed to demonstrate the impact of high versus low expression of these core genes on HCC patient prognosis. ROC curves were employed to assess the diagnostic performance of these core genes for HCC. The relationship between key prognostic genes and HCC immune cell infiltration was analyzed. Finally, molecular docking was utilized to further investigate the interactions between key prognostic genes and BPA.

**Results:**

A total of 101 intersecting genes were identified in BPA and HCC by multi-database analysis.GO and KEGG functional enrichment analysis showed that these intersected genes could affect HCC progression through multiple pathways.Five prognostic core genes, SRC, PPARG, HSP90AA1, MAPK3 and ESR1, were differentially expressed in HCC and were associated with poor prognosis of HCC patients. The ROC curves showed that the five prognostic core genes had good diagnostic and predictive properties. Meanwhile, an immune infiltration analysis suggested that the five prognostic genes had an important regulatory role in the immune microenvironment of HCC. In addition, molecular docking analysis further confirmed the potential interaction between BPA and the five prognostic core genes.

**Conclusion:**

The results suggest that SRC, PPARG, HSP90AA1, MAPK3 and ESR1 play crucial roles in the development of HCC promoted by BPA. This provides new theoretical insights into the molecular mechanisms by which BPA promotes disease progression in HCC and offers potential therapeutic targets for early diagnosis, prognostic assessment, and targeted therapy for HCC.

## Highlights

• We identified 101 intersecting genes linking Bisphenol A exposure to hepatocellular carcinoma.
• Five core genes (SRC, PPARG, HSP90AA1, MAPK3, ESR1) are prognostic biomarkers for HCC.
• BPA stably binds to all five core genes, as validated by molecular docking.
• The core genes are significantly involved in hormone response and nuclear receptor pathways.
• This study reveals novel molecular bridges between an environmental pollutant and HCC progression.

## 1 Introduction

Studies have reported that the epidemiological burden of Hepatocellular carcinoma (HCC), one of the leading causes of cancer-related deaths, is projected to continue to increase over the next three decades [1]. Additionally, new risk factors (e.g., environmental exposures) have emerged [2], which have a significant impact on the early detection, diagnosis, and treatment of HCC. Studies have shown that environmental exposures play a potential role in the rising incidence of HCC [3], indirectly contributing to HCC through their genotoxicity and promotion of progressive liver fibrosis and cirrhosis [4]. Unfortunately, the overall impact of the environment on HCC has not yet been thoroughly assessed, and the underlying mechanisms remain elusive. Bisphenol A (BPA), a lipophilic compound widely used in the manufacture of plastics, resins, food packaging bags, and other industrial manufacturing, has become ubiquitous in both the environment and living organisms, and human exposure to BPA has gradually increased [5]. Numerous studies have shown that BPA is potentially biotoxic, causing histopathological changes or dysfunctions related to a variety of cell signalling pathways in cardiovascular disease [6], reproductive system [7] [8], diabetes [9], obesity, immune dysfunction, and malignant tumours such as HCC [10]. Some studies have confirmed that even very low levels of BPA exposure may have an impact on human health or increase the risk of cancer (e.g., HCC, colorectal, breast, prostate) [11] [12], and that BPA may regulate tumour growth, proliferation, migration, invasion, apoptosis, and even drug resistance through a variety of signalling pathways [13].

**Fig. 1:**
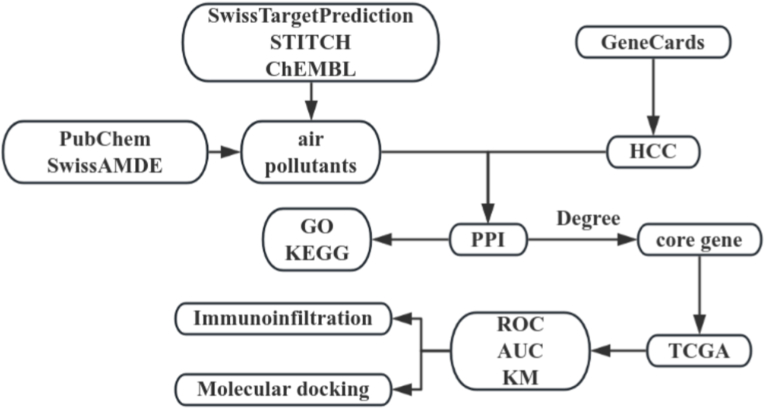
Research work flow chart.

Network toxicology, a derivative field developed from network pharmacology, overcomes the limitations of traditional studies [14] and helps to construct a complex regulatory network of chemical exposure-toxicity target-disease pathway [15], which is a cutting-edge approach for target discovery and toxicity mechanism studies, while molecular docking studies can further improve the predictive accuracy of toxicological assessment [16]. To fill the aforementioned research gap, the aim of this study was to for the first time employ network toxicology, combined with molecular docking, to systematically analyse the relationship between BPA-associated prognostic key genes and HCC progression. Specifically, we integrated multiple public databases to construct a comprehensive protein-protein interaction network, identified and prioritized core hub genes, and performed molecular docking. This integrated approach helps to elucidate the systemic mechanism of BPA-induced HCC, provide novel insights into the role of BPA in HCC development, and lay a foundation for subsequent experimental verification and targeted intervention. Fig. 1 shows the detailed workflow of the study.

## 2 Materials and methods

### 2.1 Collection of BPA and HCC-associated genes

SMILES ID numbers of BPA were obtained from PubChem (https://pubchem.ncbi.nlm.nih.gov) and SwissAMDE database (http://www.swissadme.ch/); SMILES ID numbers were used to screen potential targets of BPA that interact only with humans in ChEMBL database (https://www.ebi.ac.uk/chembl/); STITCH (http://stitch.embl.de/) and SwissTargetPrediction database (http://www.swisstargetprediction.ch/) were supplemented with BPA potential targets, and the final BPA gene set was obtained after merging and removing duplicates. HCC-associated genes were obtained by entering "Hepatocellular carcinoma" in the GeneCards database (https://www.genecards.org/), and HCC-associated target genes with relevance score ≥ median were retained after downloading the data. All database searches were conducted with filters restricted to "*Homo sapiens*".

### 2.2 Acquisition of intersecting genes and PPI network construction

BPA and HCC-associated target genes were screened and merged by Venny 2.1.0 online webpage (https://bioinfogp.cnb.csic.es/tools/venny/index.html), and the intersecting genes were taken and plotted as Wayne diagrams; PPI analyses of the intersecting genes was performed using the STRING database (https://cn.string-db.org/), and set the confidence score threshold ≥0.4 to select biologically significant interactions; and then visualise the results by Cytoscape 3.9.1 software to construct the PPI network.

### 2.3 GO and KEGG enrichment analysis

GO and KEGG pathway enrichment analyses of the intersecting genes was performed using the DAVID (https://davidbioinformatics.nih.gov/tools.jsp) and KOBAS (http://bioinfo.org/kobas/genelist/) databases. For GO enrichment analysis, all three GO categories were explicitly analyzed, including Biological Process (BP), Molecular Function (MF), and Cellular Component (CC). The statistical criteria applied for identifying significantly enriched GO terms and KEGG pathways were a significance threshold of p-value <0.05 and a q-value (FDR-adjusted p-value) < 0.05. The analysis revealed the potential roles of the intersected genes in the biological processes, molecular functions, and cellular components of HCC, as well as the signalling pathways they are involved in, and uncovering and visualising toxicant-disease target pathways and regulatory mechanisms.

### 2.4 Selection of core genes and validation by bioconfidence analysis

Core genes were identified using the CytoHubba plugin in Cytoscape, with the Degree algorithm applied as the screening criterion. Genes ranked in the top 10 by this algorithm were selected as core genes. Specifically, the CytoHubba plug-in was used to screen out the top 10 core genes from the intersected genes using Degree as the criterion, and the expression differences of the 10 core genes in HCC and healthy controls were further verified by the TCGA database (https://portal.gdc.cancer.gov); the KM survival curves were plotted to further demonstrate the differential 5 core genes with poor prognosis in HCC patients; demonstrated the correlation between prognostic core genes using correlation heatmap; verified the predictive accuracy efficacy of core genes for HCC using receiver operating characteristic (ROC) curve and area under the curve (AUC) values; finally, the ssGSEA algorithm was used to calculate the immune infiltration of the core genes and 24 immune cells in HCC, and a lollipop plot was drawn to visualise the results.

### 2.5 Molecular docking

The PDB files of the five prognostic core genes and BPA were retrieved from the RCSB database (https://www.rcsb.org/) and downloaded; the molecular docking analyses of each of the five prognostic core genes and BPA were performed using the CB-Dock2 online website (https://cadd.labshare.cn/cb-dock2/index.php) [17], following the standard protocol of the tool as described in its original publication. To ensure methodological transparency and reproducibility, key parameters and procedures of the docking analysis are explicitly reported: cavity detection was performed using CB-Dock2's integrated solvent-accessible surface clustering algorithm with the default distance cutoff of 4 Å, where candidate binding pockets were automatically ranked by solvent-accessible surface area (SASA) and hydrophobicity score; docking calculations were executed via the embedded AutoDock Vina engine with its native scoring function (evaluating hydrogen bonds, hydrophobic interactions, electrostatic forces, and van der Waals contacts) to generate binding energy values (kcal/mol); ligand poses were ranked in ascending order of binding energy, with poses exhibiting binding energy ≤ −5 kcal/mol defined as stable binding conformations, and the top-ranked pose (lowest binding energy) for each protein-ligand complex was selected for subsequent analysis and visualization, consistent with the standard criteria of CB-Dock2 and relevant bioinformatics studies [17].

## 3 Results

### 3.1 Acquisition of BPA and HCC-associated genes

A total of 148 BPA-associated genes were obtained from ChEMBL, STITCH, and SwissTargetPrediction databases. A total of 9669 HCC-associated genes were retrieved from GeneCards database. To optimise the dataset, the median Relevance score was calculated to be 5.972, and retained a total of 5669 HCC-associated genes for all Relevance score ≥ median.

### 3.2 BPA-HCC intersection gene and PPI network construction

**Fig. 2:**
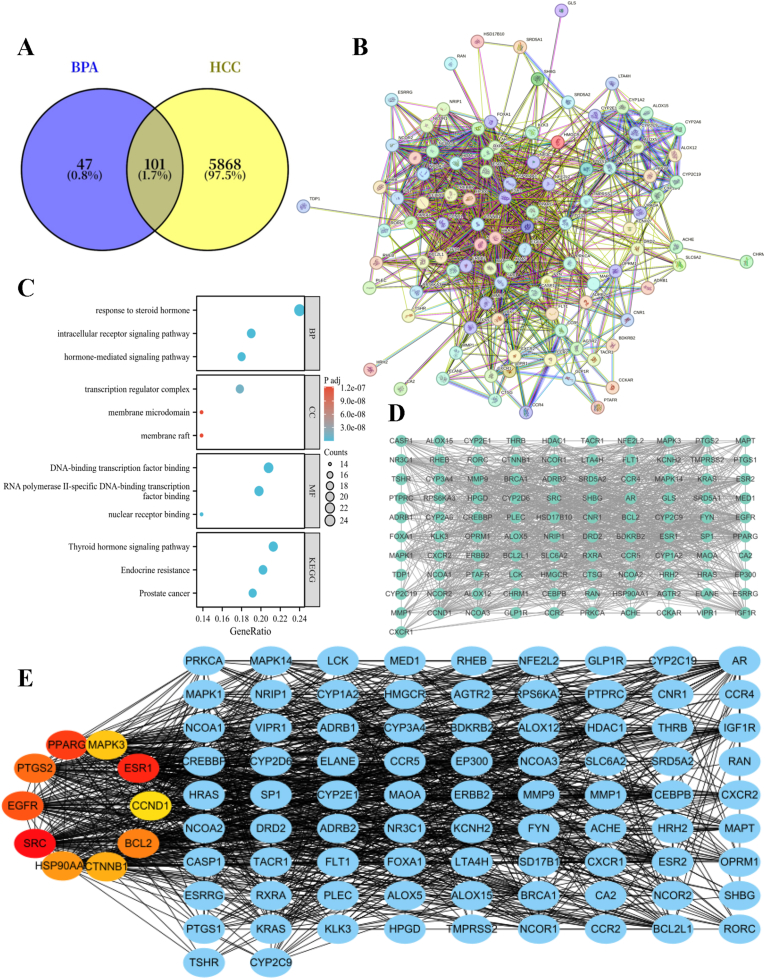
Acquisition and selection of genes related to BPA and HCC. (A) Venn diagram; (B) PPI network diagram; (C) GO and KEGG pathway enrichment analysis diagram; (D) Intersecting gene diagram; (E) Core gene diagram.

To analyse the association between BPA and HCC, the 148 BPA-associated genes obtained were taken to intersect with the 5669 HCC-associated genes obtained, and a total of 101 intersected genes were obtained (Fig. 2 A). The 101 intersected genes were imported into the STRING database for PPI analysis, and the confidence score threshold was set ≥0.4 (Fig. 2 B). The 101 intersected genes were further imported into Cytoscape 3.9.1 software for visualising the PPI network (Fig. 2 D).

### 3.3 GO and KEGG pathway enrichment analysis

**Fig. 3:**
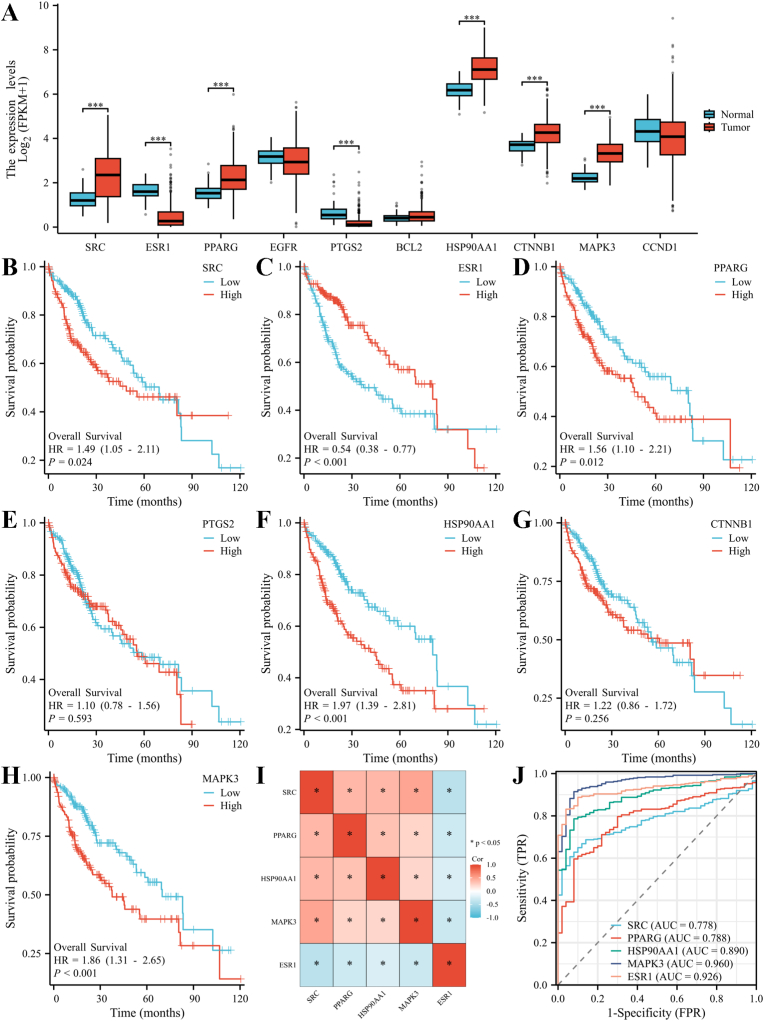
Core gene selection and validation. (A) Differences in the expression levels of the 10 initially screened core genes between HCC tissues and normal healthy liver tissues; (B–H) KM survival curves of 7 core genes (SRC, ESR1, PPARG, PTGS2, HSP90AA1, CTNNB1, and MAPK3); (I) Correlation heatmap of the 5 key prognostic-related core genes (SRC, PPARG, HSP90AA1, MAPK3, and ESR1); (J) ROC curves of the 5 key prognostic-related core genes (SRC, PPARG, HSP90AA1, MAPK3, and ESR1), with the AUC values used to verify the diagnostic predictive efficacy of these core genes for HCC.

GO and KEGG pathway enrichment analysis were performed on the above 101 intersected genes, and the GO functional enrichment analysis showed that the 101 intersected genes were involved in a total of 144 biological processes (BP), 29 cellular components (CC) and 110 molecular functions (MF), which are significantly enriched for molecular functions mainly related to biological processes such as steroid hormone response, cellular components such as transcriptional regulatory complexes, and DNA-binding transcription factor binding. KEGG pathway enrichment analysis showed that the 101 intersected genes were involved in 176 signalling pathways, which were mainly enriched in metabolic and disease signalling pathways involving cell growth and death, signal transduction, genetic information processing and other functional categories. Fig. 3 C shows the top three most significantly enriched pathways in GO, KEGG. This indicates that BPA-HCC intersected genes are significantly enriched in transcription factor binding, nuclear receptor binding, and other molecular functions, suggesting that the proteins encoded by these intersected genes mainly play key roles at the transcriptional level, and are effector molecules at the downstream of hormone signalling pathways, whose core functions lie in interfering with intracellular hormone (especially steroid hormones) signalling, abnormally activating or inhibiting the downstream nuclear receptor signalling pathways, which in turn lead to the disruption of the downstream gene transcriptional regulatory network. This disruption at the molecular level not only disrupts normal endocrine function but also disrupts the hepatic signaling regulatory network, which is a specific manifestation of the core molecular mechanism of BPA as an endocrine disruptor for carcinogenesis in HCC.

### 3.4 Selection of core genes

The top 10 core genes were screened by applying Degree algorithm through CytoHubba plug-in, including SRC, ESR1, PPARG, EGFR, PTGS2, BCL2, HSP90AA1, CTNNB1, MAPK3, and CCND1 (Fig. 2 E). The differences in the expression of the above 10 core genes in HCC and normal tissues were verified by TCGA database. The results showed that ESR1 and PTGS2 were lowly expressed in HCC, SRC, PPARG, HSP90AA1, CTNNB1, and MAPK3 were highly expressed in HCC, whereas the differences between EGFR, BCL2, and CCND1 were not statistically significant in HCC and normal tissues (Fig. 3 A). The KM survival curves showed that among the seven genes with differential expression, high expression of SRC, PPARG, HSP90AA1, and MAPK3 was associated with poor patient prognosis (Fig. 3B–D,F,H), low expression of ESR1 was associated with poor patient prognosis (Fig. 3 C), while PTGS2 and CTNNB1 had no statistically significant difference with patients' prognosis (Fig. 3E–G). The heatmap of the five prognosis-associated genes showed that there was a varying degree of correlation between the gene pairs (Fig. 3 I), which revealed potential synergistic or antagonistic effects between genes and provided clues for in-depth study of the mechanisms of gene regulatory networks in the development of HCC.

### 3.5 Diagnostic and prognostic value of core genes in HCC

The ROC curves showed that five prognosis-related core genes, including SRC (AUC = 0.778), PPARG (AUC = 0.788), HSP90AA1 (AUC = 0.890), MAPK3 (AUC = 0.960), and ESR1 (AUC = 0.926), all had good efficacy for the diagnosis of HCC (all with AUC>0.7). Among them, ESR1 had the highest AUC value and relatively best diagnostic accuracy (Fig. 3 J), and is recommended as an independent biomarker for clinical prediction of prognosis in HCC patients.

### 3.6 Immune infiltration

**Fig. 4:**
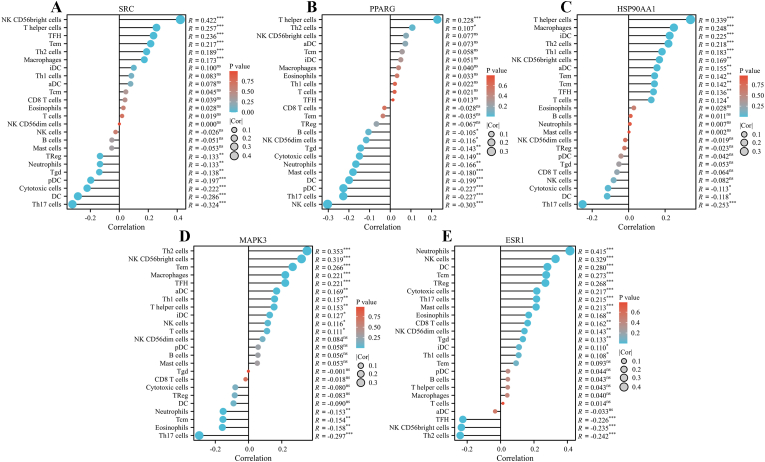
Analysis of immune infiltration between five core genes related to prognosis (SRC, PPARG, HSP90AA1, MAPK3 and ESR1) and 24 immune cells in HCC tissues. (A) SRC gene and immune cell infiltration correlation; (B) PPARG gene and immune cell infiltration correlation; (C) HSP90AA1 gene and immune cell infiltration correlation; (D) MAPK3 gene and immune cell infiltration correlation; (E) ESR1 gene and immune cell infiltration correlation.

The above five core genes associated with HCC prognosis were subjected to immune infiltration analysis. The results showed different degrees of correlation between the different genes and various types of immune cells, which implied that they played important roles in the immune microenvironment of HCC, providing new clues for exploring the development of HCC, the mechanism of immune escape, and searching for potential therapeutic avenues (Fig. 4A–E).

### 3.7 Molecular docking

**Fig. 5:**
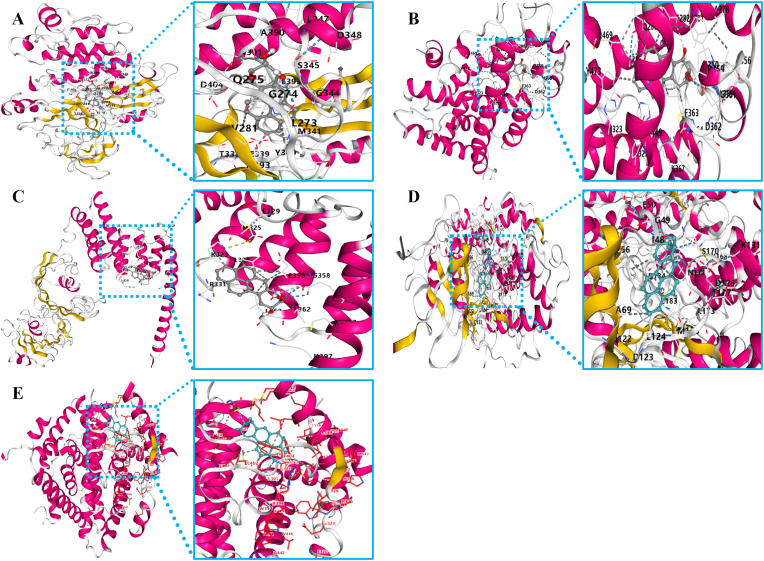
Molecular docking diagram between BPA and five key prognostic core genes (SRC, PPARG, HSP90AA1, MAPK3 and ESR1). (A) Molecular docking diagram of BPA and SRC protein; (B) Molecular docking diagram of BPA and PPARG protein; (C) Molecular docking diagram of BPA and HSP90AA1 protein; (D) Molecular docking diagram of BPA and MAPK3 protein; (E) Molecular docking diagram of BPA and ESR1 protein.

Molecular docking analysis showed that the binding energies of the five prognostic core key genes to BPA were all ≤ −5 kcal/mol (Table 1), which suggests that the five prognostic core key genes can spontaneously and stably bind to BPA (Fig. 5A–E). This suggests that BPA can directly interact with key genes in HCC patients, which in turn affects the development of HCC.

**Table 1:** Binding energy between BPA and core genes.

	SRC	PPARG	HSP90AA1	MAPK3	ESR1
Binding energy	−9.7	−8.2	−7.6	−10.6	−9.1

## 4 Discussion

HCC, as the main type of primary liver cancer, is one of the leading causes of cancer-related deaths globally, and its epidemiological burden is extremely high and continues to rise.Traditional risk factors such as chronic hepatitis B virus (HBV) infection, chronic alcohol abuse, non-alcoholic fatty liver disease and hepatitis-induced cirrhosis have been widely recognized as major drivers of HCC development.However, in recent years there has been growing evidence that environmental exposures, as an emerging and increasingly important risk factor, play a key role in the rising incidence of HCC. Among the many environmental chemicals that may influence the occurrence of HCC, BPA has received particular attention due to its widespread presence and potential biotoxicity.In 1936, Dowds and Lawson discovered that BPA, with estrogen (ES)-like properties, is a typical endocrine disrupting chemicals (EDC) [18], which binds to a variety of nuclear receptors in the human body, especially through the estrogen receptor (ESR), and also interacts with receptors for androgens and thyroid hormones [19]. BPA may directly or indirectly promote the growth, proliferation, migration, and invasion of liver tumour cells, inhibit their apoptosis, and may even affect tumour resistance to treatment by regulating multiple signalling pathways. In this study, we found that among the top 10 Hub genes screened: high expression of SRC, PPARG, HSP90AA1, and MAPK3 was associated with poor prognosis of patients, and low expression of ESR1 was associated with poor prognosis of patients, and these five core genes had better efficacy in the diagnosis of HCC, and may be used as biomarkers for the diagnosis of HCC.

GO analysis suggested that the three most significant GO-enriched terms were: steroid hormone response, intracellular receptor signalling pathway, and hormone-mediated signalling pathway, and that nuclear receptor binding and its downstream events (DNA-binding transcription factor binding, and RNA polymerase II-specific transcription factor binding) were significantly enriched, suggesting that nuclear receptor-driven transcriptional regulation is a central mechanism of BPA toxicity. KEGG pathway enrichment analysis suggested a significant enrichment of the classical nuclear receptor pathways, the thyroid hormone signalling pathway and endocrine resistance, suggesting that BPA not only activates pro-mitotic nuclear receptor signalling, but also induces therapeutic resistance.Notably, prostate cancer is a typical hormone-dependent cancer, and the enrichment of the prostate cancer pathway suggests that the hormonal signalling pathways interfered by BPA in HCC may be related to the shared mechanisms of hormone-dependent cancers, further emphasizing the centrality of the hormonal pathways in BPA-caused HCC.Taken together, BPA aberrantly activates or inhibits its downstream nuclear receptor signalling pathways mainly through endocrine disrupting mechanisms, disrupting the hepatic transcriptional regulatory network, and thus promoting the development of HCC.

ESR1, a pivotal nuclear receptor mediating estrogen (ES) signaling, plays a central role in regulating reproductive functions, participating in cell differentiation and proliferation, and maintaining tissue homeostasis, primarily through the classical ES signaling pathway [20]. The liver, a major site of ES metabolism, is precisely regulated by ES signaling, particularly via ESR1. ES can inhibit the progression of HBV-associated HCC by reducing HBV RNA transcription and inflammatory factor levels [21]. As a modulator of the tumor immune response [22], it suppresses HCC cell proliferation and invasiveness by influencing genomic effector pathways, oncogene expression, and remodeling the immune microenvironment. Notably, ESR1-mediated ES signaling is closely implicated in regulating immune cell infiltration within the HCC microenvironment—specifically, it modulates the recruitment and activation of tumor-associated macrophages (TAMs), T lymphocytes, and natural killer (NK) cells, which are core components of the anti-tumor immune response. BPA, as an EDC with a phenolic structure similar to ES, can competitively bind to estrogen receptors (ESRs), mimicking endogenous ES [23] [24], and this binding not only disrupts normal endocrine functions and hepatic signaling networks but also impairs the ESR1-dependent regulation of immune cell infiltration. Specifically, BPA interference may skew TAM polarization toward the M2 pro-tumor phenotype, reduce cytotoxic T cell infiltration, and inhibit NK cell activation, thereby weakening anti-tumor immunity and creating an immune-suppressive microenvironment that facilitates HCC progression. This provides a clear biological link between BPA exposure, ESR1 dysregulation, immune cell infiltration disorders, and HCC development, highlighting ESR1's role in BPA-mediated immune system modulation in HCC. BPA exposure may impair cellular function by inducing oxidative stress and excessive reactive oxygen species (ROS) production, leading to DNA damage, apoptosis, and oxidative damage to proteins and lipids [25,26]. Zhu et al. [27] found that BPA exposure increased methylation in steroidogenic gene promoter regions, inhibiting gene transcription, and thereby affected steroid hormone synthesis.

Sarcoma (SRC), the first discovered proto-oncogene, is a non-receptor tyrosine kinase that plays a pivotal role in regulating cell growth, migration, and survival-key processes dysregulated in BPA-induced HCC [28]. Notably, the SRC signalling pathway is not only critical for cancer cells to evade apoptosis and promote survival but also involved in chemical carcinogenesis (via receptor activation), lipid metabolism disorders, and atherosclerosis, all of which are closely linked to BPA-induced toxicity and HCC progression [29] [30]. Importantly, SRC signaling is a key regulator of immune cell infiltration and function in the HCC microenvironment: it modulates the adhesion, migration, and activation of immune cells (including T cells, macrophages, and neutrophils) by regulating the expression of chemokines and adhesion molecules. BPA, as an endocrine-disrupting chemical, exerts its carcinogenic effects partly by aberrantly activating key signalling pathways through receptor interactions, and our findings highlight that SRC is a critical mediator of this process in HCC-with additional implications for immune system modulation. Molecular docking results further support BPA exhibited stable binding to the SRC protein. This binding likely interferes with SRC's normal kinase activity, thereby disrupting its downstream signalling cascades—including the regulation of YAP nuclear translocation (which enhances transcriptional activity to promote HCC cell proliferation, migration, and invasion) and PARP1 phosphorylation (which induces resistance to PARP1 inhibitors in HCC cells) [31]. Moreover, BPA-induced SRC dysregulation may disrupt the balance of immune cell infiltration: aberrant SRC activation can promote the recruitment of M2 TAMs and myeloid-derived suppressor cells (MDSCs), while inhibiting the infiltration of cytotoxic CD8^+^T cells, thus fostering an immune-suppressive microenvironment that accelerates HCC progression. This deepens our understanding of SRC's role in BPA-mediated HCC development by linking its dysregulation to immune system modulation, which is a key biological implication of our findings. Importantly, SRC and PARP1 have been recognized as a "synthetic lethal" target pair for HCC therapy [32], and our findings suggest that BPA exposure may disrupt this target pair's balance by interfering with SRC function, further exacerbating HCC progression and treatment resistance. This interference not only promotes HCC cell proliferation, migration, and invasion but also contributes to treatment resistance, underscoring the critical role of SRC in the integrated mechanism of BPA-induced HCC and providing a novel insight into potential therapeutic strategies targeting BPA-related HCC.

Peroxisome Proliferator-Activated Receptor Gamma (PPARG) is a key transcription factor that plays a central role in regulating lipid metabolism, adipose differentiation, insulin sensitivity, and inflammatory responses—processes closely intertwined with BPA-induced chemical carcinogenesis, lipid disorders, and atherosclerosis, contribute to HCC progression [33]. Beyond its metabolic functions, PPARG is increasingly recognized as a critical modulator of immune cell infiltration in the HCC microenvironment: it regulates the expression of immune-related genes and chemokines, thereby influencing the recruitment and polarization of TAMs, T cells, and B cells. PPARG activation typically promotes anti-inflammatory and immune-suppressive phenotypes, which can be hijacked by tumor cells to evade anti-tumor immunity, and BPA exposure may amplify this effect through direct binding to PPARG. BPA exhibited stable binding to PPARG in our molecular docking results. This direct binding likely interferes with PPARG's normal transcriptional activity, thereby dysregulating its downstream cascades—including the promotion of lipid synthesis, enhancement of pro-tumorigenic effects via Akt2 signalling activation [34], and stabilization by Ubiquitin Specific Peptidase 22 (USP22) to further boost fatty acid synthesis and HCC progression [35]. Notably, BPA-induced PPARG dysregulation may skew the immune microenvironment toward immunosuppression by increasing M2 TAM polarization, reducing CD4^+^T and CD8^+^T cell infiltration, and upregulating the expression of immune checkpoints, thereby weakening anti-tumor immune responses and promoting HCC progression. This clarifies the biological implication of PPARG in BPA-mediated immune system modulation in HCC, linking PPARG dysregulation, immune cell infiltration disorders, and BPA-induced hepatocarcinogenesis. This interference promotes lipid synthesis-driven tumorigenesis and exacerbates HCC development, highlighting PPARG's key role in the integrated mechanism of BPA-induced HCC and providing new insights into targeting lipid metabolism pathways for BPA-related HCC intervention.

HSP90AA1 encodes Heat shock protein 90α (Hsp90α), a highly conserved molecular chaperone widely expressed in eukaryotic cells that plays a pivotal role in maintaining cellular homeostasis [36]. Critically, Hsp90α is closely linked to chemical carcinogenesis, lipid metabolism disorders, and atherosclerosis—key pathological processes that contribute to HCC progression [37]. As an endocrine-disrupting chemical, BPA exerts its hepatocarcinogenic effects partly by interfering with the function of key regulatory proteins like Hsp90α, which in turn dysregulates downstream oncogenic cascades. Importantly, Hsp90α is a key mediator of immune cell infiltration and function in the HCC microenvironment: it regulates the stability and activity of immune-related proteins that control the production of pro-inflammatory cytokines and chemokines, thereby influencing the recruitment of TAMs, neutrophils, and T cells. Dysregulated Hsp90α function—induced by BPA binding—can disrupt the balance of immune cell infiltration, favoring the formation of an immune-suppressive microenvironment. molecular docking results demonstrated that BPA form stable bindings with Hsp90α. This direct binding is hypothesized to interfere with Hsp90α′s normal chaperone function, thereby enhancing its ability to stabilize a variety of oncoproteins (e.g., kinases, transcription factors) that buffer cancer cell stress and promote malignant transformation [38]. Furthermore, BPA-induced Hsp90α dysregulation may promote the recruitment of M2 TAMs and MDSCs, inhibit cytotoxic T cell activation, and upregulate pro-inflammatory cytokines that further exacerbate immune dysregulation and HCC progression. This reveals the critical biological implication of HSP90AA1 in BPA-mediated immune system modulation in HCC, linking BPA exposure, Hsp90α dysfunction, immune cell infiltration disorders, and hepatocarcinogenesis. Notably, this dysregulation may also reinforce the AFP-HSP90-Myc/Met signalling axis (where AFP binds Hsp90α to stabilize downstream oncoproteins like Myc and Met), further exacerbating HCC development [39]. This finding underscores HSP90AA1 as a promising therapeutic target for BPA-related HCC.

Mitogen-activated protein kinase 3 (MAPK3), also known as extracellular regulatory protein kinase 1 (ERK1), is a key member of the MAPK kinase family that plays a critical role in integrating upstream signalling cascades. Importantly, MAPK3 is closely associated with chemical carcinogenesis, lipid metabolism disorders, and atherosclerosis [40]. As an immune-ferroptosis-related gene (IFRG) [41], MAPK3 is intimately involved in regulating immune cell infiltration and anti-tumor immune responses in the HCC microenvironment—its dysregulation directly impacts the recruitment, activation, and function of cytotoxic T cells, NK cells, and TAMs, which are key to controlling tumor progression. BPA-induced MAPK3 dysregulation may thus disrupt immune homeostasis in HCC, providing a direct link between BPA exposure, immune system modulation, and hepatocarcinogenesis. Molecular docking results demonstrated that BPA form stable bindings with MAPK3. This direct binding to interfere with MAPK3's normal function as a downstream effector of the Raf/MEK/ERK pathway, thereby amplifying signalling during tumour invasion and metastasis by dysregulating cell proliferation, differentiation, and survival [40]. Notably, MAPK3 as an immune-ferroptosis-related gene (IFRG), where its upregulation may induce an immune-suppressive microenvironment that weakens anti-tumour immune responses, further exacerbating HCC progression and poor prognosis [41]. Specifically, BPA-induced MAPK3 overactivation can promote the secretion of immunosuppressive cytokines and chemokines that recruit M2 TAMs and MDSCs, while inhibiting the infiltration and activation of CD8^+^T cells and NK cells—this creates an immune-suppressive microenvironment that facilitates HCC cell escape from anti-tumor immunity. This deepens the biological implication of MAPK3 in BPA-mediated immune system modulation in HCC, clarifying how BPA-induced MAPK3 dysregulation alters immune cell infiltration to drive HCC progression. Additionally, MAPK3-mediated Ras/MAPK pathway dysregulation—likely amplified by BPA interference—plays a pivotal role in controlling HCC cell proliferation and malignant progression [42]. This interference promotes HCC invasion, metastasis, and immune suppression, highlighting MAPK3's key role in the integrated mechanism of BPA-induced HCC and providing new insights into targeting MAPK3-mediated pathways for BPA-related HCC intervention.

Molecular docking analyses showed good binding affinity and interactions between all five prognostic core key genes and BPA. This suggests that BPA can interact directly with key genes in HCC patients and that BPA exposure may be an environmental trigger for the development of HCC, which emphasises the potential environmental risk factors for the development of HCC. In this study, we applied network toxicology and molecular docking techniques to explore the toxic role and potential molecular pathways of the environmental pollutant BPA in the development of HCC, which provided a theoretical basis for an in-depth discussion of the biological mechanisms by which BPA promotes HCC, and a scientific basis for limiting the exposure to BPA in daily life.

## 5 Limitations and future research directions

It is critical to explicitly acknowledge the limitations of the present study, which is based primarily on bioinformatics prediction and public data mining, relying on network toxicology models and molecular docking techniques—common inherent constraints of computational research in toxicology and oncology. Specifically, the core data originated from public database mining, which may be subject to potential database biases. Such biases could affect the reliability and generalizability of our bioinformatics analyses, as the inherent variability in public data may not fully reflect the actual molecular landscape of BPA-induced HCC in diverse populations or experimental settings. Meanwhile, molecular docking analyses adopted in this study are static in nature and cannot fully reflect dynamic molecular interactions in living organisms—they only predict the binding affinity and conformation between BPA and the five core genes, but fail to capture real-time changes in binding dynamics, post-translational modifications, or the influence of the complex intracellular microenvironment on these interactions. Additionally, the in silico approaches employed are difficult to completely simulate the complex environmental and molecular regulatory networks in vivo, including the crosstalk between the five core genes, the dynamic regulation of immune cell infiltration, and the interplay between BPA exposure, endocrine disruption, and hepatocarcinogenesis—all of which are critical for understanding the actual biological mechanisms underlying BPA-induced HCC. Most importantly, no experimental validation was performed to verify the predicted results, such as in vitro cell experiments or in vivo animal models, which are essential to confirm the functional roles of the five core genes and their regulatory effects on immune cell infiltration in BPA-induced hepatotoxicity and HCC progression. Therefore, our findings should be interpreted with caution, and it is still necessary to carry out further experimental validation on the basis of the present study. We anticipate that future experimental studies will provide more direct biological evidence to support our predictions, thereby offering experimental support for further elucidating the mechanism of BPA-induced hepatotoxicity and HCC development. Overall, the results of this study provide a valuable hypothesis-generating framework that requires future experimental confirmation to fully validate the proposed molecular mechanisms.

## 6 Conclusion

In this study, we integrated network toxicology and molecular docking to screen and validate five core prognostic genes (SRC, PPARG, HSP90AA1, MAPK3, ESR1) that mediate BPA-induced HCC development. These genes are differentially expressed in HCC tissues, associated with poor patient prognosis and possess good HCC diagnostic efficacy. Functional enrichment analysis showed they are mainly involved in steroid hormone response and intracellular receptor signaling pathways, and immune infiltration analysis confirmed their key regulatory roles in the HCC immune microenvironment via modulating immune cell recruitment and polarization. Molecular docking verified stable binding between BPA and all five core proteins (binding energy ≤ −5 kcal/mol), indicating BPA may disrupt their normal functions, interfere with hepatic endocrine and immune homeostasis, and thus promote HCC progression. Our findings identify these five genes as key molecular links between BPA exposure and HCC carcinogenesis, clarify the potential molecular and immune regulatory mechanisms of BPA-induced hepatotoxicity, and provide novel candidate targets for early diagnosis, prognostic assessment and targeted intervention of BPA-related HCC. Subsequent in vitro and in vivo experiments are required to verify the functional roles and specific regulatory mechanisms of these core genes in BPA-induced HCC, so as to translate these bioinformatics predictions into clinical practice.

## Author contributions statement

Y.N.Z and Y.W wrote and designed the manuscript; Y.N.Z and Y.W acquired the data; C.J.W. and Y.X.H improved the figure quality; Y.J.Z and Z.H.Z participated in a discussion and made language edits; X.F.Z and J.C.Z. revised and reviewed the manuscript. All authors read and approved the manuscript.

## Ethical statement

Not applicable.

## Funding

Tutor Program at 10.13039/501100012562Gansu University of Traditional Chinese Medicine (2023YXKY020); Lanzhou Youth Science and Technology Talents Innovation Project (2024-QN-39).

## Declaration of competing interest

All authors declare that they have no conflict of interest.

## Data Availability

Data will be made available on request.
